# Menopause-Specific Quality of Life among Emirati Women

**DOI:** 10.3390/ijerph17010040

**Published:** 2019-12-19

**Authors:** Linda Smail, Ghufran Jassim, Anam Shakil

**Affiliations:** 1 Department of Mathematics and Statistics, Zayed University, Dubai 19282, UAE; M80007064@zu.ac.ae; 2 Royal College of Surgeons, Ireland-Medical University of Bahrain, Busaiteen 15503, Bahrain; gjassim@rcsi-mub.com

**Keywords:** menopause, quality of life, Emirati women, MENQOL, vasomotor

## Abstract

To investigate the quality of life (QOL) of menopausal Emirati women aged 40–64 years and determine its relationship with their sociodemographic characteristics. A community-based cross-sectional study was conducted on 70 Emirati women using multistage stratified clustered random sampling. The participants were interviewed face-to-face using a structured questionnaire comprising sociodemographic variables, reproductive characteristics, and the Menopause-Specific Quality of Life (MENQOL) questionnaire. The most common symptom among the study participants was ‘aching in the muscles’. The participants had a moderate level of bothersome symptoms; in addition, vasomotor symptoms were reported by 61%, while sexual symptoms were only reported by one-third of the participants. There were no significant differences between the menopausal status in any of the four domains of the MENQOL questionnaire. Additionally, there were no significant differences between the mean scores of the four MENQOL domains and all predictors. This study highlights the importance of educating women about menopause and its symptoms.

## 1. Introduction

Menopause is a transitional process experienced by over 500 million women between the ages of 45 and 55 years each year worldwide [1]. This number is expected to increase to 1200 million women by the year 2030. Menopause refers to the time when a woman’s menstrual period stops for 12 consecutive months after the last period and is characterized by ovarian failure, thus leading to a decline in the production of the ovarian hormones estrogen and progesterone. The lack of these hormones makes women prone to experiencing common symptoms that include, but are not limited to, sleep disorders, mood alterations, hot flashes, depression, urinary tract infections, vaginal infections, and increased risk for osteoporosis and cardiovascular diseases. Apart from these physical symptoms, such women are also inclined to experience several vasomotor, somatic, sexual, and psychological symptoms, thereby affecting their quality of life (QOL) [2]. The World Health Organization (WHO) defines QOL as “an individual’s perception of their position in the context of the culture and value systems in which they live and in relation to their goals” [3]. QOL includes six domains: physical health, social relationships, psychological state, spiritual concerns, environmental features, and level of independence [4]. Improving and maintaining a good QOL is a very important health goal not only for menopause-related medicine but also for governments and health care authorities [5].

Quality of life is perceived as a major health component, especially for menopausal women, and has become an essential research topic that has been discussed widely in the literature [5].

Wilson and Clearly [6] have illustrated the relationships between cultural attitudes, environmental changes, hormonal changes, menopausal symptoms, change in family composition, and declining health status. In fact, personal motivation and attitudes towards menopause amplify the severity of the symptoms. Additionally, social and psychological support and even economic support help women relieve the burden of menopause [7]. As reported by Fuh et al. [8], Fallahzadeh [9] and Abedzadeh et al. [10], it can be observed that all these different factors contribute to the QOL of women during and after menopause.

Educating women about menopause, its management, and the use of menopausal hormone therapy (MHT) may serve as an important step towards treating postmenopausal symptoms [11], thereby improving the QOL of postmenopausal women. According to a recent study, the level of awareness and knowledge of women is directly proportional to their ability to control their symptoms [12].

Women’s health is looked upon differently in various cultures, even though the need for knowledge and information in this area is crucial. Thus, conducting a study that investigates the intimate areas of women’s health, such as MHT and menopause and its symptoms, is very important, especially in the Arab world. In the Arab culture, menopause is often characterized by a social stigma among most women because it signifies the end of reproduction and hence is referred to as the ‘age of desperation’. Women perceive menopause to be a natural phase in which menstruation ends and women are no longer able to conceive. The main concern is the inability to conceive and aging, not the bothersome symptoms or the associated risks that come along with these factors. This is partly due to the lack of education and knowledge of the symptoms during menopause and partly due to the impact imposed by the family and spouse regarding fertility. This notion results in a major limitation in regard to using MHT to treat menopausal symptoms, thereby making it extremely challenging for physicians to control these symptoms because of the limited effectiveness of alternative nonhormonal options.

The available literature on the QOL of menopausal women is primarily derived from Asian and industrialized Western populations. There is currently very little evidence derived from Arab countries and an extreme paucity in regard to data on the QOL of menopausal women in the United Arab Emirates. In fact, no studies exploring the QOL of Emirati menopausal women have been conducted in the Emirate of Dubai. The proposed study will be the first to investigate the QOL of Emirati menopausal women in the Emirate of Dubai. This study will add more information about Emirati menopausal women to the limited QOL and menopause research in the Arab world, specifically in the United Arab Emirates.

## 2. Materials and Methods

### 2.1. Study Design and Sample

A community-based cross-sectional study was conducted among a random sample of 70 Emirati women aged 40 to 64 years from the Emirate of Dubai. The study participants were recruited from five primary health care (PHC) centers in Dubai.

### 2.2. Sampling Method

The public health care system in Dubai consists of twenty health care centers and peripheral clinics representing twenty regions throughout the Emirate of Dubai [13]; each region is represented by one health center. A random sample of 70 Emirati women aged 40–64 years were enrolled in the study via a multistage stratified and clustered random sampling technique. The first stage was a clustered random sample in which five health centers were chosen to participate. The second stage was a stratified random sample based on age, in which two age groups were identified (40–49 and 50–64). The final stage was a simple random sample from each age group from the five chosen health centers. The sample size was determined based on a power of 85% and a significance level of 5%. The study was carried out from April 2018 to August 2018. Women with serious health problems, such as malignant tumors or serious chronic diseases, were not included in the study.

### 2.3. Study Instruments

A structured questionnaire was administered through face-to-face interviews in the PHC centers. The questionnaire included two parts. The first part assessed the sociodemographic variables and reproductive characteristics, and the second part contained the Menopause-Specific Quality of Life (MENQOL) questionnaire, which was developed by Hilditch et al. [7]. In the first section, participants were asked about their education level, employment status, marital status, age group, smoking habits, use of MHT, use of oral contraceptive pills, health status, and menopausal status (i.e., premenopause, perimenopause or after menopause [postmenopausal]).

In the second part of the questionnaire, the participants were asked to indicate whether they had experienced symptoms or problems within the past month and, if so, to rate how bothersome they were on a seven-point Likert scale from 0 to 6: with 0 indicating that they were not at all bothered and 6 indicating that they were extremely bothered.

The 29 questions of the MENQOL were divided into four domains: vasomotor (items 1–3), psychosocial (items 4–10), physical (items 11–26), and sexual (items 27–29).

### 2.4. Validity and Reliability of the Questionnaire

The questionnaire was translated into the Arabic language by a professional translator and was further checked by a bilingual speaker before being translated back to the original language, i.e., English. A panel of experts from the same specialty evaluated the content for suitability, language simplicity and clarity in reading. Their comments were taken into consideration, and necessary amendments were made to the questionnaire. This assessment was performed via a pilot study of 12 women who often visited a particular PHC center. A measurement of 0.941 on the MENQOL was derived based on Cronbach’s method. The main study was exclusive to the women who participated in the pilot study.

### 2.5. Ethical Consideration

The research and ethics committee at Zayed University approved the research on ethical grounds. The Dubai Health Authority and the chosen PHC centers also gave their approval for the data collection associated with this study.

### 2.6. Statistical Analysis

The collected data were coded, entered, and analyzed using the statistical package SPSS version 25 (IBM Corp, Armonk, New York, NY, USA). Descriptive statistics were computed to analyze all items from the questionnaire. For the analysis, we converted the score of each item to a score ranging from 1 to 8. The seven-point Likert scale from 0–6 was converted to a 7-point score ranging from 2 to 8; a score of 1 was given when participants answered ‘No’, indicating that they had not experienced the symptoms at all in the past month. Thus, the scores ranged from 1 to 8.

We computed the mean (range, 1 to 8) and the standard deviation for each domain. The overall MENQOL score is the mean of all 4 domains. We reported means, standards deviations, percentages, and confidence intervals in each case.

The designed study had three categories for menopause defined as follows:

1. Premenopause: Having regular periods.
2. Perimenopausal: Changes in periods but have not gone 12 months in a row without a period, have not stopped completely, or have occasional spotting.
3. Postmenopausal: No period at all.

Due to the low numbers representing the premenopause category, we identified two categories: Perimenopausal (pre- and perimenopausal combined together) and Postmenopausal, defined as follows:

1. Perimenopausal: Pre- and perimenopausal: women who had had regular menstrual periods within the last 3 months or who indicated that their periods had become irregular, but they had had a period in the last 12 months.
2. Postmenopausal: women who indicated that they had not had a period in the last 12 months or longer

This status was determined by asking the participants about the number of months since their last menstrual period.

Sociodemographic and reproductive variables served as independent variables, while the four domains of the MENQOL were considered dependent variables. One-way analysis of variance (ANOVA) or the independent-sample t-test were carried out to test the differences in population means across the categories of each independent variable (predictor) based on the independent variable category. Statistical tests with *p*-values < 0.05 were considered statistically significant.

## 3. Results

A total of 70 women participated in this study, and the mean age (±SD) was 53.4 (±7.8) years, with a range of 40 to 64 years. The median age was 55 years. (Table 1). Table 2 shows the sociodemographic characteristics of the participants by menopausal status.

Table 3 shows the frequency and percentages of the women’s answers to the MENQOL questionnaire. The most frequent symptom was ‘aching in the muscles and joints’ at 78.6%, and the least reported symptom was ‘being dissatisfied with my personal life’ at 32.9%.

Table 4 shows the mean scores for all MENQOL domains and the overall scores by menopausal status. The mean of all four domains was 3.27, with a range of 1 to 7.18. This mean score was less than the average mean, indicating a moderate level of bothersome symptoms, which indicates that women were able to cope with their menopause symptoms. It is clear that among all participants, the psychosocial and sexual domains showed the lowest mean scores, while the physical and vasomotor domains showed the highest mean scores. Low scores indicated a better QOL. Vasomotor symptoms were reported by 61% of participants, while sexual symptoms were reported by only 33.3%.

By comparing the overall mean score and the mean scores of the domains among Emirati women by their menopausal status, it was found that the postmenopausal women had a significantly lower mean than the perimenopausal women in the four domains, with very low mean scores in the psychosocial and sexual domains and a lower overall mean score. Thus, postmenopausal women had a better QOL than perimenopausal women.

Table 5 shows the Spearman correlation coefficients between the four domains, which indicates that there was a highly significant correlation between the four domains at a significance level of 0.01.

No significant differences in the mean scores on the four MENQOL domains for each of the predictor variables (education level, smoking, marital status, employment and age) were observed.

## 4. Discussion

Menopause is considered an important experience that changes a woman’s life in different aspects; therefore, the QOL of menopausal women is important to assess. In our study, we found that the most commonly reported symptoms were physical in nature: aches in the muscles and joints (at 78.6%) and backaches (at 75.7%). Most of the other physical symptoms were also commonly experienced. This finding is similar to what was reported by Khadija et al. [14] regarding Pakistani women who reported that the most common symptoms were low backache (at 85.2%) and vaginal dryness (at 84.3%). The most reported symptoms in the study conducted by Bener et al. [15] were aches in the back of the neck or head (at 46.4%) and aches in the muscles and joints (at 34.6%). Additionally, Mahajan et al. [16] reported that fatigue, or ‘being tired’, was the most commonly reported symptom (62%) among Indian women.

These findings differ from those in most European and Western studies in which the most commonly reported symptoms were hot flashes rather than other physical symptoms [17,18]. This difference may be explained by the different cultures; for example, in Japan, the menopause phase is called ‘konenki’, which means ‘renewal years and energy’ [19], while the Arabic phrase for the menopause phase is ‘desperate age’ because it is considered the end of women’s life as they are no longer reproductive. Therefore, cultural beliefs may account for the diversity in how menopausal women experience and cope with menopause symptoms [20].

Similarly to our results, in the study by Nisar and Sohoo [21], it was found that among all four domains, the physical domain was more significantly associated with postmenopausal women and that menopause had a negative impact on the QOL of the participants (Iranian women).

In our study, vasomotor symptoms were reported by 61% of the participants, psychosocial symptoms were reported by 48.8% of the participants, physical symptoms were reported by 42.3% of the participants, and sexual symptoms were reported by only 33.3% of the participants. This is similar to what was reported in the study conducted in the United Arab Emirates by Bener et al. [15]; in fact, in their study, employed women experienced more symptoms. Based on the four domains, 69% of participants reported physical symptoms, 58.7% reported psychosocial symptoms, 40% reported vasomotor symptoms and 37.9% reported sexual symptoms. The low reporting of symptoms in the sexual domain is similar to what has been reported by studies, such as [22,23], conducted in other countries. This may be related to culture, as discussing sexual problems is considered taboo in many cultures, including the Arabic one.

The reported low mean QOL score, 3.27 (±1.62), of women in this investigation, indicates a low severity of bothersome symptoms and therefore a moderate QOL of the Emirati women involved in the study. This finding is higher than the reported mean QOL score of Saudi women [24], which was 2.74 (±1.43), indicating an even lower QOL. However, while in our study the psychosocial and sexual domains showed the lowest mean scores, while the physical and vasomotor domains showed the highest mean scores, the corresponding findings were completely the opposite in [24]. In fact, the results regarding Saudi women showed that sexual and psychosocial domains had the highest mean scores, while vasomotor and physical domains had the lowest mean scores.

This difference may be explained by the fact that in the Saudi study, 53% of the participants (47 out of the 90 participants) had a university level education, as compared to only 25% of the Emirati women. This may be because educated Saudi women felt more comfortable talking about sexual symptoms and Emirati women did not. Another factor that may explain the difference in the results despite the similar culture is that the Saudi women involved in the study did not use MHT at all, while in our sample, more than 11% of Emirati women declared using or having used MHT in the past.

In this study, women experienced moderate menopausal symptoms, and perimenopausal women scored higher on the questionnaire than postmenopausal women. This is expected because postmenopausal women have already gone through this phase and are no longer experiencing the symptoms or are experiencing less severity of them. Additionally, unmarried women had higher mean scores (lower QOL) in the vasomotor and physical domains, but the differences were not statistically significant. Women with a primary school education level had higher scores in all domains. Employed women had higher mean scores than unemployed women. Women who smoked had higher mean scores (lower QOL) in the vasomotor domains. This may be explained by the fact that perimenopausal women answered the questionnaire on the basis of their knowledge and not their experience.

Additionally, Nisar and Sohoo [21] showed that the mean score in the psychosocial domain was higher in the menopausal group than in the postmenopausal group, which is consistent with the findings of our study.

In this study, no significant differences between QOL and the predictor variables (education level, smoking, marital status, employment and age) were observed. In contrast, Narozi et al. [25] found that the QOL of postmenopausal women was related to their age, education level, marital status, and employment status. Additionally, in the study by Abedzadeh et al. [10], only education level and employment status influenced the QOL of Iranian women.

## 5. Strengths and Limitations of the Study

One important strength of our study is that it is the first study to investigate the QOL of Emirati menopausal women in the Emirate of Dubai. Our study was limited by the small sample size. This was explained by the gynecologists working in the PHC centers as being due to the fact that Emirati women consult them only in emergencies, and if the women do seek medical care, they only consult female doctors. Because of the limited number of participants in the present study, it was unfeasible to categorize them by age group. Other limitations of our study included the descriptive design, the absence of a comparison group, and recall bias. Choosing an interview instead of a self-administered survey is a double edge sword. Whereas the interview provided a consistent and clear description of the questions, a self-administered survey would have allowed more space and freedom to answer sensitive questions.

## 6. Conclusions

While the sample size is relatively small and cannot not be considered fully representative of all menopausal Emirati women, the results provide insights and a better understanding of the QOL of Emirati women and related factors. This study showed that Emirati women experienced moderate menopausal symptoms and that the most commonly reported symptoms were physical in nature. It also reported that menopausal women had a better QOL than postmenopausal women.

As the life expectancy of Emirati women has increased, they are expected to live at least one-third of their lives beyond menopause. Experiencing menopause symptoms has negative effects on the QOL of women in general; therefore, there is a need to assess menopausal women’s QOL and to consider it a major public health issue.

We herein highlight the importance of educating women about menopause, its symptoms, the long-term risks and the pros and cons of MHT. Health care providers should consider the QOL of menopausal women and continuously assess their needs.

## Figures and Tables

**Table 1:** The sociodemographic characteristics of the participants.

Variable	Level	*N*	%
Educational level	Primary School	19	27.1
	Preparatory School	13	18.6
	Secondary School	20	28.6
	University Graduate	18	25.7
Employment	Yes	14	20
	No	56	80
Marital status	Unmarried	24	34.3
	Married	46	65.7
Menopausal status	Perimenopausal	18	25.7
	Postmenopausal	52	74.3
Smoking	Yes	4	5.7
	No	65	92.9
Perceived health status	Not Good	11	15.7
	Good	44	62.9
	Very Good	15	21.4
Use of oral contraceptive pills	Used	30	42.9
	Never used	39	55.7
Use of menopausal hormone therapy	Current users	5	7.1
	Past users	3	4.3
	Never used	62	88.6
Age by group	[40–49]	16	22.9
	[50–64]	53	75.7
Time since menopause in years	Minimum = 1	Mean	SD
	Maximum = 20	6.3	5.2

**Table 2:** The sociodemographic characteristics of the participants by menopause category.

Variable	Perimenopausal (*N* = 18) n (%)	Postmenopausal (*N* = 52) n (%)
**Educational Level**		
Primary School	6 (33)	13 (25)
Preparatory School	3 (17)	10 (19)
Secondary School	6 (33)	14 (27)
University Graduate	3 (17)	15 (29)
**Employment**		
Yes	14 (78)	42 (81)
No	4 (22)	10 (19)
**Marital status**		
Unmarried	7 (39)	16 (31)
Married	11 (61)	35 (69)
**Smoking**		
Yes	0 (0)	4 (8)
No	18 (100)	48 (82)
**Age**	47.94 ± 10.55	55.27 ± 5.49

**Table 3:** Percentages of participants who answered yes to experiencing menopause symptoms.

Symptoms	Perimenopausal *N* (%)	Postmenopausal *N* (%)	Total *N* (%)
Hot flashes	13 (18.6)	35 (50)	48 (68.6)
Nights sweats	12 (17.2)	29 (41.4)	41 (58.6)
Sweating	9 (12.9)	30 (42.8)	39 (55.7)
Being dissatisfied with their personal life	10 (14.3)	13 (18.6)	23 (32.9)
Feeling anxious or nervous	13 (18.6)	30 (42.8)	43 (61.4)
Experiencing poor memory	12 (17.2)	29 (41.4)	41 (58.6)
Accomplishing less than they used to	8 (11.4)	22 (31.5)	30 (42.9)
Feeling depressed, down or blue	11 (15.7)	27 (38.6)	38 (54.3)
Being impatient with other people	12 (17.2)	25 (35.7)	37 (52.9)
Feelings of wanting to be alone	8 (11.4)	19 (27.2)	27 (38.6)
Flatulence or gas pains	13 (18.6)	32 (47.7)	45 (64.3)
Aches in the muscles and joints	15 (21.4)	40 (57.2)	55 (78.6)
Feeling tired or worn out	10 (14.3)	36 (51.4)	46 (65.7)
Difficulty sleeping	11 (15.7)	34 (48.6)	45 (64.3)
Aches in the back, neck or head	12 (17.2)	41 (58.5)	53 (75.7)
Decrease in physical strength	9 (12.9)	33 (47.1)	42 (60.0)
Decrease in stamina	11 (15.7)	25 (35.7)	36 (51.4)
Feeling a lack of energy	12 (17.2)	35 (49.9)	47 (67.1)
Dry skin	10 (14.3)	33 (47.1)	43 (61.4)
Weight gain	12 (17.2)	33 (47.1)	45 (64.3)
Increased facial hair	9 (12.9)	22 (31.4)	31 (44.3)
Changes in appearance, texture or tone of skin	7 (10)	21 (30)	28 (40.0)
Feeling bloated	11 (15.7)	27 (38.6)	38 (54.3)
Low backache	12 (17.2)	37 (52.8)	49 (70.0)
Frequent urination	12 (17.2)	24 (34.2)	36 (51.4)
Involuntary urination when laughing or coughing	9 (12.9)	28 (40)	37 (52.9)
Change in sexual desire	10 (14.3)	24 (34.3)	34 (48.6)
Vaginal dryness during intimacy	13 (18.6)	27 (38.5)	40 (57.1)
Avoiding intimacy	8 (11.4)	18 (25.7)	26 (37.1)

**Table 4:** Descriptive statistics of the MENQOL domains.

Domain	Perimenopausal Mean (SD) 95% CI	Postmenopausal Mean (SD) 95% CI	MENQOL Mean (SD)	%	*p*-Value
Vasomotor	3.70 (2.11) (2.79, 4.62)	3.26 (1.87) (2.72, 3.79)	3.37 (1.93)	61	0.410
Psychosocial	3.72 (1.93) (2.84, 4.61)	2.83 (1.87) (2.31, 3.35)	3.06 (1.91)	48.8	0.087
Physical	3.73 (1.82) (2.95, 4.50)	3.57 (1.59) (3.11, 4.02)	3.61 (1.64)	42.3	0.725
Sexual	3.56 (2.15) (2.53, 4.58)	2.85 (2.18) (2.25, 3.45)	3.03 (2.18)	33.3	0.236
All domains	3.67 (1.87) (2.92, 4.44)	3.12 (1.52) (2.68, 3.57)	3.27 (1.62)	56.3	0.215

**Table 5:** Spearman’s correlation matrix: the calculation was based on the presence/absence of symptoms.

Domain	Vasomotor	Psychosocial	Physical	Sexual
Vasomotor	1.000	0.799 *	0.523 *	0.495 *
Psychosocial	0.799 *	1.000	0.614 *	0.647 *
Physical	0.523 *	0.614 *	1.000	0.410 *
Sexual	0.495 *	0.647 *	0.410 *	1.000

* Correlation is significant at the 0.01 level (2-tailed).
